# The effect of model structure and data availability on Usutu virus dynamics at three biological scales

**DOI:** 10.1098/rsos.231146

**Published:** 2024-02-07

**Authors:** Nora Heitzman-Breen, Yuganthi R. Liyanage, Nisha Duggal, Necibe Tuncer, Stanca M. Ciupe

**Affiliations:** ^1^ Department of Mathematics, Virginia Polytechnic Institute and State University, Blacksburg, VA, USA; ^2^ Department of Mathematical Sciences, Florida Atlantic University, Boca Raton, FL, USA; ^3^ Department of Biomedical Sciences and Pathobiology, VA-MD College of Veterinary Medicine, Virginia Tech, Blacksburg, VA, USA

**Keywords:** mathematical model, virus dynamics, multi-scale models, model identifiability, immuno-epidemiological models

## Abstract

Understanding the epidemiology of emerging pathogens, such as Usutu virus (USUV) infections, requires systems investigation at each scale involved in the host–virus transmission cycle, from individual bird infections, to bird-to-vector transmissions, and to USUV incidence in bird and vector populations. For new pathogens field data are sparse, and predictions can be aided by the use of laboratory-type inoculation and transmission experiments combined with dynamical mathematical modelling. In this study, we investigated the dynamics of two strains of USUV by constructing mathematical models for the within-host scale, bird-to-vector transmission scale and vector-borne epidemiological scale. We used individual within-host infectious virus data and per cent mosquito infection data to predict USUV incidence in birds and mosquitoes. We addressed the dependence of predictions on model structure, data uncertainty and experimental design. We found that uncertainty in predictions at one scale change predicted results at another scale. We proposed *in silico* experiments that showed that sampling every 12 hours ensures practical identifiability of the within-host scale model. At the same time, we showed that practical identifiability of the transmission scale functions can only be improved under unrealistically high sampling regimes. Instead, we proposed optimal experimental designs and suggested the types of experiments that can ensure identifiability at the transmission scale and, hence, induce robustness in predictions at the epidemiological scale.

## 1. Introduction

Usutu virus (USUV) is an emerging zoonotic flavivirus similar to West Nile virus (WNV) [1,2] that circulates in sub-Saharan Africa, central Europe and the Mediterranean basin [3–5]. It is maintained in the environment through an enzootic cycle involving mosquitoes and birds; and has been associated with decreased bird populations in Europe, occasional spillover to mammals, including humans [2,6], and a few cases of neurological complications in humans [2,7,8]. Given the novelty of the USUV, it is of great importance to understand how individual-scale processes impact population-scale spread. Since the virological and epidemiological patterns are usually independent of each other, mathematical modelling can serve as a tool to link knowledge at these scales. To better understand the bird–mosquito USUV transmission cycle, we investigate the within-host viral dynamics inside an infected bird, the bird-to-mosquito transmission probability and the epidemiological-level bird and mosquito infection incidence.

In previous work, we used within-host mathematical models to study the viral–host interactions of different USUV strains following individual bird (juvenile chicken) infections and found that the European USUV strains (*Spain 2009* and *Netherlands 2016*) have higher replication rates and peak viremia compared with the African USUV strains (*Uganda 2012* and *South Africa 1959*) [9,10]. A recent empirical study has shown that birds (wild-caught house sparrows and juvenile chickens) inoculated with *Netherlands 2016* have higher probability of infection to mosquito (*Culex quinquefasciatus*) than birds inoculated with *Uganda 2012* [11].

In this study, we investigate the relationship between USUV strain characteristics and per cent mosquitoes infection, and use that inference to assist how model and data structure at these scales impact model predictions of USUV incidence in bird and mosquito populations. To achieve this, we develop an age-structured vector-borne disease model and parametrize it using *Netherlands 2016* and *Uganda 2012* longitudinal infectious viral titres in infected birds combined with bird-to-mosquito transmission data [11]. We use the model to predict the mechanistic interactions that describe USUV incidence dynamics in bird and mosquito populations. The paper also aims to determine whether scarcity of data used in validating within-host models and bird-to-vector probability of infection functions results in biases in the predicted disease incidence. To address this, we investigate structural and practical identifiability of the within-host model and practical identifiability of the bird-to-vector probability of infection functions and propose solutions for improvement in predictions through addition of extra and/or alternative measurements.

## 2. Material and methods

### 2.1. Within-host scale

**Figure 1.**
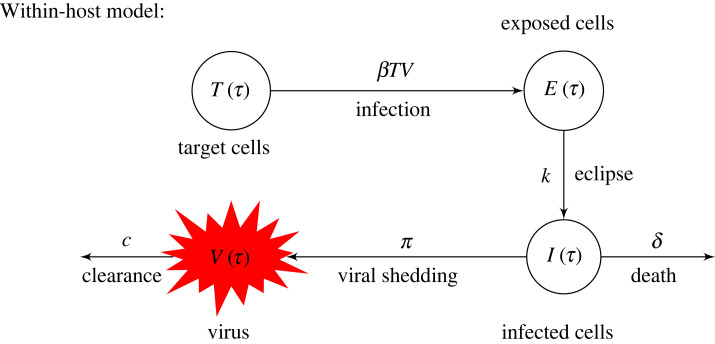
Model diagram describing the within-host USUV infection.

*Within-host model.* We consider a within-host model with eclipse phase used in other acute viral infections [9,10,12,13] to describe the within-host USUV dynamics. The model includes uninfected leukocytes *T*, exposed leukocytes *E*, productively infected leukocytes *I* and USUV *V*. We investigate their dynamics *τ* days after infection, as follows [9]. Following viral infection, target cells become exposed at rate *β* and exposed cells become productively infected cells at rate *k*. Productively infected cells produce *π* virions per day and die at rate *δ*. Virus is cleared at rate *c*. Given the short timespan of USUV infection within an infected bird (around 7 days) no renewal or death terms are considered for the leucocyte populations. A diagram describing these interactions is given in figure 1 and the model is described by the following equations:

2.1 $$\begin{array}{rl} & \frac{dT}{d\tau }=-\beta TV,\\ & \frac{dE}{d\tau }=\beta TV-kE,\\ & \frac{dI}{d\tau }=kE-\delta I,\\ & \frac{dV}{d\tau }=\pi I-cV, \end{array}\}$$

with initial conditions *T*(0) = *T*_0_, *E*(0) = 0, *I*(0) = 0, *V*(0) = *V*_0_. Note that the effect of viral loss through cell entry, given by −*βTV*, is negligible compared with virus production and clearance terms and, therefore, not included in the virus equation. We aim to estimate model equation (2.1)’s parameters by fitting it to published infectious virus titres in wild-caught house sparrows infected with either *Netherlands 2016* or *Uganda 2012* [11].

*Empirical data.* Data consists of two cohorts (*n* = 14 each) of wild-caught house sparrows, confirmed to be seronegative for WNV, and subcutaneously inoculated with 1500 plaque forming units (PFU) of USUV *Netherlands 2016* or *Uganda 2012*. Infectious viral titres (in PFU ml^−1^) $V_{\tau }^{d}$ were collected daily for the first 7 days post inoculation *τ* = {1, 2, …, 7}. Of the *n* = 14 birds inoculated with *Netherlands 2016* only *n* = 11 had virus measured above the limit of detection (LOD = 100 PFU ml^−1^) and only *n* = 10 had at least two viral titre measurements above the limit of detection. All *n* = 14 birds inoculated with *Uganda 2012* had viremia above the limit of detection, but only *n* = 6 had at least two viral titres above the limit of detection [11].

*Data fitting algorithm.* We assume an initial target population $T(0)=4\times 10^{6}\;\mathrm{cells}\;ml^{-1}$ [10] and no exposed or infected cells $E(0)=I(0)=0\;\mathrm{cells}\;ml^{-1}$. The empirical infectious inoculum was 1500 PFU which, when distributed across 2.5 ml of blood [14], gives an initial inoculum concentration of 600 PFU ml^−1^. We assumed an initial $V(0)=10\;\mathrm{PFU}\;ml^{-1}$ infectious titre, and rejected $V(0)=100\;\mathrm{PFU}\;ml^{-1}$ and $V(0)=600\;\mathrm{PFU}\;ml^{-1}$ initial conditions through model selection (see table S1 in the electronic supplementary material). All other parameters **p** = {*β*, *δ*, *c*, *π*, *k*} are unknown. We only use the birds that have at least two data points above limit of detection, *n* = 10 and *n* = 6 for *Netherlands 2016* and *Uganda 2012* infection, respectively. We exclude bird U-Sp8 from *Uganda 2012* fits due to unrealistic data fitting predictions (see electronic supplementary material, figure S1). Hence, the number of birds inoculated with *Uganda 2012* that are used in this study becomes *n* = 5.

We estimate the mean, median and standard deviation of population parameters **p** using a nonlinear mixed effects modelling approach that uses stochastic approximation estimation–maximization (SAEM) algorithm in Monolix [15]. Briefly, we assume that population parameters **p** are lognormally distributed with mean ln(μ) and standard deviation ***σ***. Moreover, we assume that the proportional error between the model and the data, $(V_{\tau }^{d}-V)/V$, is normally distributed with mean zero and standard deviation *η*. Since several data measurements are below the limit of detection (LOD = 100 PFU ml^−1^), we consider these measurements to be censored data. They are incorporated into the data fitting in Monolix by assuming that each censored data point takes a value between zero and the LOD [16,17]. Besides mean population parameters, we also estimate individual bird parameters **p**_*i*_ (for bird *i*) using the Nelder–Mead (NM) simplex algorithm in Monolix software [15]. Model equation (2.1)’s goodness of fit, given by the Akaike information criterion (AIC) index, is

AIC=−2LL(p)+2N,

where *LL* is the log-likelihood and *N* is the number of estimated parameters in the nonlinear mixed effects model (in our case *N* = 11, 10 for the individual mean and standard deviations of the five fitted parameters and one for the population standard deviation).

### 2.2. Bird-to-mosquito transmission scale

*Bird-to-mosquito probability of infection mathematical formulation.* To determine the relationship between the USUV load inside a bird *V*(*τ*) and the probability of a transmission event to a mosquito at day *τ*, we develop three models describing the probability of bird-to-mosquito transmission as functions of viral load *V*(*τ*). We assume the number of infectious viruses in the sample that establish an infection in a mosquito is a random variable that follows a Poisson distribution with mean *E*[*V*]. If each transmissible virus has a probability *ρ* of establishing infection in a mosquito, then the number of viruses that are successfully transmitted follows a Poisson distribution with parameter *λ* = *E*[*V*]*ρ* [18] and the probability of one or more viruses successfully infecting a mosquito is

2.2 b(V)=1−exp(−λ).

We consider the following three models for the mean transmissible virus *E*(*V*) [19],

1. *The linear model* assumes that the mean transmissible virus is proportional to the detectable viral load (above the limit of detection), i.e.
  $$E_{1}(V)=\nu (V-D),$$
  where $D=\mathrm{LOD}=100\;\mathrm{PFU}\;ml^{-1}$ and *ν* is a positive constant. The corresponding probability of infection becomes
  2.3 $$b_{1}(V)=1-\exp(-a(V-D)),$$
  where *a* = *νρ*.
2. *The power-law model* assumes that the mean transmissible virus is proportional to the power of birds’s detectable viral load, i.e.
  $$E_{2}(V)=\nu {\left(V-D\right)}^{h},$$
  where *ν* and *h* are positive constants. The corresponding probability of infection becomes
  2.4 $$b_{2}(V)=1-\exp(-a{(V-D)}^{h}),$$
  where *a* = *νρ*.
3. *The density-dependent model* assumes that the mean transmissible virus is proportional to a density-dependent function of the bird’s detectable viral load, expressed by a Hill-type function, i.e.
  $$E_{3}(V)=\nu \frac{{\left(V-D\right)}^{h}}{{\left(V-D\right)}^{h}+L^{h}},$$
  where *L* is the above limit of detection bird’s viral load where the growth is half-maximal and *ν* and *h* are positive constants. The corresponding probability of infection becomes
  2.5 $$b_{3}(V)=1-\exp\left(-a\frac{{\left(V-D\right)}^{h}}{{\left(V-D\right)}^{h}+L^{h}}\right),$$
  where *a* = *νρ*.

*Empirical data.* *Culex quinquefasciatus* mosquitoes were fed on birds infected with USUV *Netherlands 2016* and *Uganda 2012*, as follows. Mosquitoes were fed on seven birds: three of the wild-caught house sparrows used in the within-host models (N-Sp1, N-Sp9 and N-Sp3) and four juvenile chickens (bred for low antibody titres) *τ* = 2 days after they were inoculated with 1500 PFU *Netherlands 2016* [11]. The paired bird viral load data (on $\log_{10}$ scale) at day 2, $\log_{10}v_{j}=\log_{10}V_{j}(2)$, and proportion of infected mosquitoes data $b_{j}^{d}$ for bird inoculated with *Netherlands 2016* is

2.6 $$\begin{array}{rl} T_{n} & =\{(\log_{10}v_{j},b_{j}^{d}),j=\{1,2,\ldots ,7\}\}\\ & =\{(2.7,0.1);(5.98,0.16);(7.17,0.6);(3.04,0.125);(3.49,0.048);(3.66,0.1364);(2,0)\}, \end{array}$$

for N-Sp1, N-Sp9, N-Sp3 and four juvenile chickens, respectively. Similarly, mosquitoes were fed on seven birds, three wild-caught house sparrows used in the within-host models (U-Sp2, U-Sp3 and U-Sp5) and four juvenile chickens (bred for low antibody titres) *τ* = 2 days after they were inoculated with 1500 PFU *Uganda 2012* [11]. The paired bird viral load data (on $\log_{10}$ scale) at day 2, $\log_{10}v_{j}=\log_{10}V_{j}(2)$, and proportion of infected mosquitoes data $b_{j}^{d}$ for bird inoculated with *Uganda 2012* is

2.7 $$\begin{array}{rl} T_{u} & =\{(\log_{10}v_{j},b_{j}^{d}),j=\{1,2,\ldots ,7\}\}\\ & =\{(2,0);(6.56,0.6);(5.23,0.182),(3.68,0.055);(3.69,0.03);(4.54,0.125);(4.76,0.1379)\}, \end{array}$$

for U-Sp2, U-Sp3, U-Sp5 and four juvenile chickens, respectively.

*Data fitting algorithm.* We fitted the probability of infection functions $b_{1}(\log_{10}v)$, $b_{2}(\log_{10}v)$ and $b_{3}(\log_{10}v)$ to both *T*_*n*_ and *T*_*u*_ per cent mosquito infection data using a least-squares approach. The following objective functional:

2.8 $$J(a,h,L)={\left(\sum_{\;j=1}^{7}b_{i}(\log_{10}v_{j})-b_{j}^{d}\right)}^{1/2},\;i=1,2,3,$$

is minimized over the (*a*, *h*, *L*) parameter space using the local optimization *fmincon*/*fminsearchbnd* algorithms in Matlab. We used the following parameter bounds: *a* ∈ [0, 10], *h* ∈ [0, 10] and *L* ∈ [0, 10].

### 2.3. Between-host scale

*Between-host model.* We considered an age-structured susceptible–infectious–recovered (SIR) epidemiological model for bird population and a susceptible–infectious (SI) epidemiological model for mosquito population. We denote the susceptible, infectious and recovered bird populations by *S*_*b*_(*t*) and *I*_*b*_(*t*, *τ*), *R*_*b*_(*t*) and the susceptible and infectious mosquito populations by *S*_*v*_(*t*) and *I*_*v*_(*t*). Time *t* represents chronological time and age *τ* represents the age of a bird infection. Mosquito infectivity rate is

**Figure 2.**
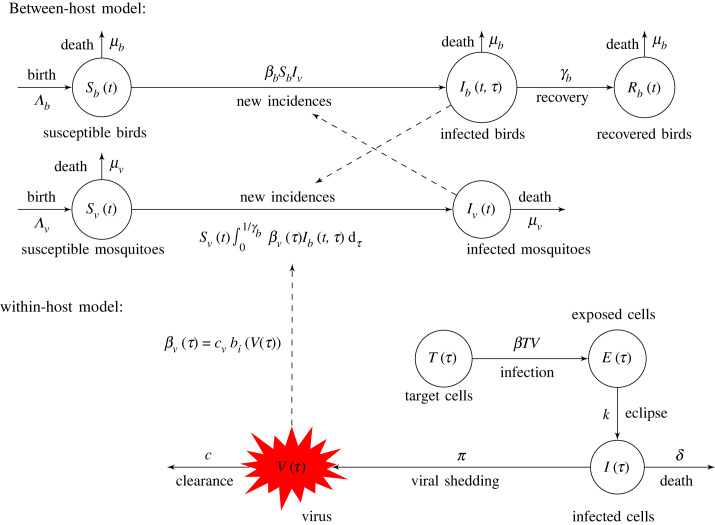
Model diagram describing the between-host USUV transmission equation (2.10).

2.9 $$\beta_{v}(\tau )=\left\{ \begin{array}{cc} c_{v}b_{i}(V(\tau )), & 0\leq \tau \leq \frac{1}{\gamma_{b}}\\ \;0, & \;\tau >\frac{1}{\gamma_{b}} \end{array}\right.$$

where *c*_*v*_ is the contact rate, *γ*_*b*_ is the bird removal rate (through recovery and/or death) and *b*_*i*_(*V*(*τ*)), *i* = {1, 2, 3} is the probability of vector infection following contact with an infected bird given by equations (2.3)–(2.5). Birds get infected through contact with a vector at constant rate *β*_*b*_ and are removed (through recovery and/or death) at constant rate *γ*_*b*_. We use a mass-action term for the force of infection, which assumes that biting rates are limited by the mosquito and bird densities. We assume a mosquito birth rate $\Lambda_{v}$ and *per capita* death rate *μ*_*v*_. Similarly, we assume a bird birth rate $\Lambda_{b}$ and *per capita* death rate *μ*_*b*_. A model description is given in figure 2 and the model equations are

2.10 $$\begin{array}{rl} & \frac{dS_{v}}{dt}=\Lambda_{v}-S_{v}\int_{0}^{1/\gamma_{b}}\beta_{v}(\tau )I_{b}(t,\tau )\;d\tau -\mu_{v}S_{v},\\ & \frac{dI_{v}}{dt}=S_{v}\int_{0}^{1/\gamma_{b}}\beta_{v}(\tau )I_{b}(t,\tau )\;d\tau -\mu_{v}I_{v},\\ & \frac{dS_{b}}{dt}=\Lambda_{b}-\beta_{b}S_{b}I_{v}-\mu_{b}S_{b},\\ & \frac{\partial I_{b}}{\partial t}+\frac{\partial I_{b}}{\partial \tau }=-\gamma_{b}I_{b}(t,\tau )-\mu_{b}I_{b}(t,\tau ),\\ & I_{b}(t,0)=\beta_{b}S_{b}I_{v}\\ \;\mathrm{and}\; & \frac{dR_{b}}{dt}=\gamma_{b}\int_{0}^{1/\gamma_{b}}I_{b}(t,\tau )\;d\tau -\mu_{b}R_{b}, \end{array}\}$$

with initial conditions $S_{v}(0)=S_{v}^{0}$, $I_{v}(0)=1-S_{v}^{0}$, $S_{b}(0)=S_{b}^{0}$, *I*_*b*_(0,* τ*) = *I*_0_, *R*_*b*_(0) = 0. We work with normalized bird and mosquito populations. Note, that while there is no recovery of infected mosquitoes, birds do recover from USUV infection. We aim to predict bird and mosquito populations' infection incidence over time if the probability of infection is described by different functions *b*_*i*_(*V*(*τ*)) given by equations (2.3)–(2.5) and if the infection is generated by *Netherlands 2016* or *Uganda 2012*.

*Parameter values.* We assume an initial 5% infected mosquitoes and 1% infected birds, i.e. *S*_*v*_(0) = 0.95, *I*_*v*_(0) = 0.05, *S*_*b*_(0) = 0.99, *R*_*b*_(0) = 0. We set *I*_*b*_(0, *τ*) = *I*_0_(*τ*) such that total infected bird population at time zero is $I_{b}(0)=\int_{0}^{1/\gamma_{b}}I_{0}(\tau )\;d\tau =0.01$. Female mosquitoes live on average one to two months [20], hence we set $\Lambda_{v}=\mu_{v}=1/60\;\mathrm{per}\;\mathrm{day}$. We set $\Lambda_{b}=\mu_{b}=1/720\;\mathrm{per}\;\mathrm{day}$, representing a house sparrow's lifespan of 2 years [21]. We fix the contact rate *c*_*v*_ = 1, the bird infectivity rate *β*_*b*_ = 0.2, and the bird recovery time 1/*γ*_*b*_ = 7 days. Under the assumed parameters, we use model equation (2.10) to compute birds' and mosquitoes' USUV incidence over time when transmission probability is *b*_*i*_(*V*(*τ*)) given by equations (2.3)–(2.5).

### 2.4. Identifiability analysis

We have defined the USUV dynamics at three different scales: within-host, bird-to-mosquito transmission and between-host. We will validate the within-host and bird-to-mosquito transmission scales with experimental data for both *Netherlands 2016* and *Uganda 2012* strains. Before estimating parameters of a mathematical model from data, we first need to study whether the model is structured to reveal its parameters from the data. This process is called the identifiability analysis. There are two types of identifiability: *structural identifiability* and *practical identifiability*. Structural identifiability describes the theoretical base for determining model parameters uniquely given the model structure and unlimited noise-free experimental observations. By contrast, practical identifiability determines whether model parameters are identifiable given limited experimental observations which are contaminated with noise.

*Structural identifiability.* Consider a model of form

2.11 $$x^{\prime }(\tau )=f\;(x(\tau ),p),$$

where *τ* denotes age of infection, *x*(*τ*) represents the vector of state variables expected to match the empirical observations of viral loads, *y*(*τ*). The goal of the structural identifiability is to determine whether model *x*′(*τ*) = *f*(*x*(*τ*), **p**) can uniquely reveal its parameters **p** given unlimited empirical observations *y*(*τ*) and no measurement errors. Several techniques have been proposed for analysing the structural identifiability of mathematical models, including those found in [22–24]. In this study, we use the differential algebra method which allows us to eliminate unobserved state variables and derive differential-algebraic polynomials of the observed variable and the model parameters, referred to as the input–output equations [24–26].

**Definition 2.1.**

Let *c*(**p**) denote the coefficients of the input–output equations where **p** is model equation (2.11)’s parameter vector. We say that model equation (2.11) is structured to reveal its parameters from the observation *y*(*τ*) if and only if

$$c(p)=c(\hat{p})\Rightarrow p=\hat{p}.$$

The structural identifiability analysis of ODE models using the differential algebra approach involves the following steps. First, model equations are transformed into a monic differential polynomial called input–output equations by eliminating unobserved state variables. The input–output equations establish the relationship between the model parameters and the observations. Next, coefficients of input–output equations are determined and the mapping from the parameter space to these coefficients is examined. If the map is one-to-one, then all parameters are identifiable, indicating that the model is structurally identifiable. If the map is not one-to-one, then the model lacks structurally identifiability. In such cases, parameter correlations are used to fix parameters, resulting in a structurally identifiable model with improved parameter estimation accuracy and reliability.

*Practical identifiability.* Structural identifiability investigates whether parameters can be estimated from a model given unlimited noise-free observations. Since, in practice, data collected is discrete and may contain significant measurement errors, structural identifiability of a model is not enough to guarantee existence of practically identifiable parameters. Therefore, it becomes necessary to determine whether structurally identifiable parameters can be estimated from noisy data. There are several methods for studying the practical identifiability of ODE models [23,24,26–31]. Here, we use the Monte Carlo simulation (MCS) approach [23,24,26,30] which follows the following steps:

1. We solve model equation (2.11) numerically using parameter values **p** obtained from fitting the model to the given discrete experimental data and then record the predictions (output) at the experimental age of infection points.
2. We generate virtual datasets by adding σ={1%,5%,10%,20%} measurement errors to each given experimental data point, with measurement errors assumed to be normally distributed with mean zero and standard deviation *σ*. For each measurement error we create *M* = 1000 such datasets.
3. For each measurement error *σ*, we fit model equation (2.11) to each of the 1000 datasets to estimate new best parameter fits **p**_*i*_, with *i* = {1, 2, …, 1000}.
4. We calculate the average relative estimation error (ARE) for each parameter of model equation (2.11)
  $$\mathrm{ARE}(p^{\left(k\right)})=100\%\times \frac{1}{M}\sum_{i=1}^{M}\frac{\left|p^{\left(k\right)}-p_{i}^{\left(k\right)}\right|}{\left|p^{\left(k\right)}\right|},$$
  where **p**^(*k*)^ is the *k*th element of the parameter set **p** and $p_{i}^{\left(k\right)}$ is *k*th element of **p**_*i*_.
5. We use the ARE formula to determine whether each parameter of model equation (2.11) is practically identifiable using definition 2.2, given below.

**Definition 2.2.**

Let ARE be the average relative estimation error of the parameter **p**^(*k*)^. The practical identifiability of parameter **p**^(*k*)^ is determined by comparing ARE with measurement error. Let ARE be smaller than a constant multiple of the measurement error *σ*, that is

$$\mathrm{ARE}(p^{\left(k\right)})<\eta \sigma .$$

(i) If 0 < *η* ≤ 1 then parameter **p**^(*k*)^ is strongly practically identifiable.
(ii) If 1 < *η* ≤ 10 then parameter **p**^(*k*)^ is weakly practically identifiable.
(iii) If 10 < *η* then parameter **p**^(*k*)^ is not practically identifiable.

A model is said to be practically identifiable when parameters **p**^(*k*)^ are practically identifiable, for all *k*.

## 3. Results

### 3.1. Within-host scale

To investigate the temporal dynamics of USUV inside infected birds we developed a within-host model equation (2.1) and fitted it to experimental data from two bird cohorts (see Material and methods).

#### 3.1.1. Structural identifiability of within-host model

Before performing data fitting, we examined the structural identifiability of the within-host model equation (2.1) (see Material and methods). We rewrite equation (2.1) in compact form

$$x^{\prime }(\tau )=f\;(x,p),$$

where *τ* denotes time since infection, *x* = {*T*, *E*, *I*, *V*} represents the vector of state variables, and **p** = {*β*, *δ*, *c*, *π*, *k*} is the vector of parameters. The model output *y*(*τ*) represent the empirical observations, which, in our case, are the viral infectious titres, *y*(*τ*) = *V*(*τ*). We compute the input–output equations using the Differential Algebra for Identifiability of System (DAISY) software [32] for equation (2.1) and obtain

3.1 $$\begin{array}{rl} 0 & =V^{⁗}V-V^{\prime \prime \prime }V^{\prime }+V^{\prime \prime \prime }V^{2}\beta +V^{\prime \prime \prime }V(c+k+\delta )-V^{\prime \prime }V^{\prime }(c+k+\delta )+V^{\prime \prime }V^{2}\beta (c+k+\delta )\\ & \;+V^{\prime \prime }V(ck+c\delta +k\delta )-V^{\prime 2}(ck+c\delta +k\delta )+V^{\prime }V^{2}\beta (ck+c\delta +k\delta )+V^{3}\beta ck\delta . \end{array}$$

According to definition 2.1 (see Material and methods), we need to show that the function mapping the parameter space to the coefficients of the input–output equation (3.1) is one-to-one. Assume that there exists another parameter vector, $\hat{p}=\{\hat{\beta },\hat{k},\hat{\delta },\hat{\pi },\hat{c}\}$, which has produced the same observation, *y*(*τ*) = *V*(*τ*). By setting $c(p)=c(\hat{p})$, we obtain

$$\beta =\hat{\beta },\;c+k+\delta =\hat{c}+\hat{k}+\hat{\delta },\;ck+c\delta +k\delta =\hat{c}\hat{k}+\hat{c}\hat{\delta }+\hat{k}\hat{\delta },\;\beta ck\delta =\hat{\beta }\hat{c}\hat{k}\hat{\delta }.$$

Solving this nonlinear system of equations in Mathematica, we obtain the following six sets of solutions

3.2 $$\begin{array}{rl} & S_{1}\;:\;\{\beta =\hat{\beta },\;c=\hat{c},\;k=\hat{\delta },\;\delta =\hat{k}\},\;S_{2}\;:\;\{\beta =\hat{\beta },\;c=\hat{c},\;k=\hat{k},\;\delta =\hat{\delta }\},\\ & S_{3}\;:\;\{\beta =\hat{\beta },\;c=\hat{\delta },\;k=\hat{c},\;\delta =\hat{k}\},\;S_{4}\;:\;\{\beta =\hat{\beta },\;c=\hat{\delta },\;k=\hat{k},\;\delta =\hat{c}\},\\ & S_{5}\;:\;\{\beta =\hat{\beta },\;c=\hat{k},\;k=\hat{c},\;\delta =\hat{\delta }\},\;S_{6}\;:\;\{\beta =\hat{\beta },\;c=\hat{k},\;k=\hat{\delta },\;\delta =\hat{c}\}. \end{array}\}$$

This reveals that only *β* is globally identifiable, whereas *c*, *k* and *δ* are locally identifiable. Lastly, parameter *π* cannot be identified as it does not appear in the input–output equation (3.1). We summarize the structural identifiability of model equation (2.1) (when we do not take into account initial conditions) in the following proposition.

**Proposition 3.1.**

*The within-host model equation* (2.1) *is not structured to reveal its parameters from the viral load* *V*(*τ*) *observations. More precisely, the infectivity rate* *β* *is globally identifiable. The eclipse rate k, killing rate δ* *and clearance rate* *c* *are locally identifiable. The viral production rate* *π*, *is not identifiable from unlimited, noise-free viral load observations* *V*(*τ*).

To acquire a better understanding of why parameter *π* is not identifiable, we scale the unobserved state variables by a positive constant ϵ>0 while keeping the observed variable unchanged, i.e. $\left\{\hat{T},\hat{E},\hat{I},V\}=\{\epsilon T,\epsilon E,\epsilon I,V\right\}$. The scaled model becomes

3.3 $$\begin{array}{rl} & \frac{d\hat{T}}{d\tau }=-\beta \hat{T}V,\;\hat{T}(0)=\epsilon T_{0},\\ & \frac{d\hat{E}}{d\tau }=\beta \hat{T}V-k\hat{E},\;\hat{E}(0)=0,\\ & \frac{d\hat{I}}{d\tau }=k\hat{E}-\delta \hat{I},\;\hat{I}(0)=0,\\ \;\mathrm{and}\; & \frac{dV}{d\tau }=\frac{\pi }{\epsilon }\hat{I}-cV,\;V(0)=V_{0}, \end{array}\}$$

which is almost identical to equation (2.1) except that the parameter *π* is scaled by 1/ϵ and initial value *T*(0) is scaled by ϵ. Without initial conditions, it is not possible to determine the scaling factor ϵ, and hence parameter *π*. When, however, all initial conditions are known, all parameters of model equation (2.1) become globally identifiable. We summarize these results, as follows.

**Proposition 3.2.**

*The within-host model equation* (2.1) *is structured to identify all its parameters from unlimited, noise-free viral load observations* *V*(*τ*) *if all initial conditions are known*.

#### 3.1.2. Within-host model dynamics

**Figure 3.**
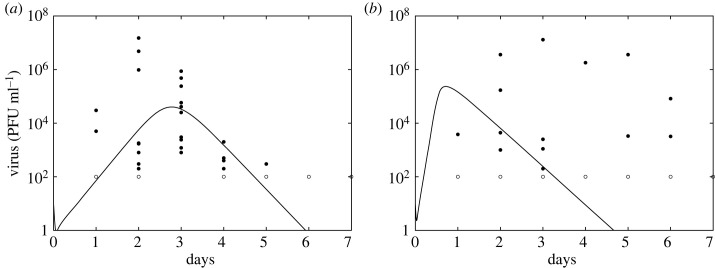
Population median virus dynamics (solid line) given by model equation (2.1) versus infectious virus titres (circles) for birds (wild-caught house sparrows) infected with (*a*) *Netherlands 2016* and (*b*) *Uganda 2012*. Model parameters are given in table 1. Note that the empty circles account for censored data.

**Figure 4.**
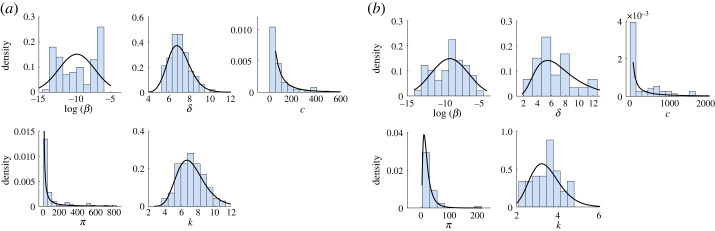
Distributions generated by simulations in Monolix (bars) versus theoretical log normal distributions of model parameters *β*, *δ*, *c*, *π* and *k* from fitting model equation (2.1) to infectious virus titre data from birds (wild-caught house sparrows) infected with (*a*) *Netherlands 2016* and (*b*) *Uganda 2012*. Mean parameter values *μ* and standard deviations *σ* are given in table 1.

As seen above, under known initial conditions and unlimited noise-free measurements, all parameters of model equation (2.1) can be identified from data. We next fitted model equation (2.1) to empirical infectious virus titre data from two bird cohorts inoculated with *Netherlands 2016* and *Uganda 2012* using a nonlinear mixed effects approach (see Material and methods). A summary of mean and standard deviations for estimated population parameters together with median parameter estimates and AIC values is given in table 1. A summary of individual estimated parameters for birds inoculated with *Netherlands 2016* is given in electronic supplementary material, table S2 and for those inoculated with *Uganda 2012* is given in electronic supplementary material, table S3. The model dynamics are plotted in figure 3 and the lognormal parameter distributions in figure 4. Model fitting results in similar population parameter estimates for infected cells death rates, $\delta =6.95\;\mathrm{da}y^{-1}$ and $\delta =6.74\;\mathrm{da}y^{-1}$ (corresponding to lifespan of 3.5 hours) for *Netherlands 2016* and *Uganda 2012* infections, respectively. The average population estimates for the infectivity and production rates, *β* and *π*, are 3 and 2.4 times higher for *Uganda 2012* compared with *Netherlands 2016*. By contrast, the average viral clearance rate, *c*, is 1.3 times lower for *Uganda 2012* compared with *Netherlands 2016*. This results in faster expansion and slower clearance for *Uganda 2012*. Lastly, the average population estimates for the eclipse phase, 1/*k*, is 3.3 and 7.1 hours for *Netherlands 2016* and *Uganda 2012*, respectively. These estimates result in average population basic reproduction number, *R*_0_ = *βp T*(0)/*cδ*, of 4.1 (range of 2.2–7) and 43 (range of 2.2–56) for *Netherlands 2016* and *Uganda 2012*, respectively. Interestingly, we find higher *R*_0_ and *π* predictions for *Uganda 2012* compared with *Netherlands 2016* in wild-caught house sparrows, which is different from higher *R*_0_ values for *Netherlands 2016* compared with *Uganda 2012* infections in juvenile chickens [9]. However, as in the juvenile chicken studies, we observe delayed clearance of *Uganda 2012* compared with clearance of *Netherlands 2016* in wild-caught house sparrows [9]. In both cohorts, we observe variability in individual viral profiles (electronic supplementary material, figures S2 and S3), and consequently differences in parameter estimates (electronic supplementary material, tables S2 and S3). This heterogeneity is larger among the birds inoculated with *Uganda 2012*, for which the number of birds was small (*n* = 5 compared with *n* = 10 for *Netherlands 2016*). For *Netherlands 2016* infections, we predict that virus peaks 2–3 days post infection (p.i.), while for *Uganda 2012* infections it peaks 0.5–6 days p.i. Virus decays below one virion ml^−1^ 4.5–7 days p.i. and 4.3–20 days p.i. for *Netherlands 2016* and *Uganda 2012*, respectively. The mean peak viremia is similar between the two strains and equal to 1.5 × 10^6^ PFU ml^−1^, and the intra cohort estimates range between 545 and 8.2 × 10^6^ PFU ml^−1^ for the *Netherlands 2016* and between 2.3 × 10^3^ and 6.2 × 10^6^ PFU ml^−1^ for *Uganda 2012*. As expected, the day of the virus peak is negatively correlated to the estimated *R*_0_ for both *Netherlands 2016* and *Uganda 2012* infections, with Pearson’s correlation coefficients −0.83 and −0.84, respectively. The magnitude of the virus peak is positively correlated to the estimated *R*_0_ in *Netherlands 2016*, with a Pearson’s correlation coefficient of 0.97. This positive correlation is not observed in *Uganda 2012* (see electronic supplementary material, figure S4).

**Table 1.** Distributions for parameters *β*, *δ*, *c*, *π* and *k* found by fitting model equation (2.1) to infectious virus titre data from birds (wild-caught house sparrows) infected with *Netherlands 2016* and *Uganda 2012*. We used the SAEM algorithm in Monolix to generate the predicted population parameter means (ln(*μ*)) and s.d. (*σ*) (see Material and methods).

		*β*	*δ*	*c*	*π*	*k*	
		$\frac{\mathrm{ml}}{\mathrm{PFU}\times d}$	$\frac{1}{d}$	$\frac{1}{d}$	$\frac{\mathrm{PFU}}{\mathrm{cell}\times d}$	$\frac{1}{d}$	AIC
*Netherlands 2016* *n* = 10	mean (*μ*)	4.66 × 10^−5^	6.95	48.8	7.49	7.07	714.1
	s.d. (*σ*)	2.63	0.16	1.21	2.66	0.24	
	median	6.4 × 10^−5^	7.14	58.7	4.56	7.43	
*Uganda 2012* *n* = 5	mean (*μ*)	1.4 × 10^−4^	6.74	35.8	18.2	3.36	407.8
	s.d. (*σ*)	2.68	0.46	2.83	0.74	0.21	
	median	2.9 × 10^−4^	6.98	50.7	17.9	3.29

We observed several sources that explain variability in parameter estimates (and unexpected predictions of higher *R*_0_ for *Uganda 2012* infection): bimodal distributions for parameter *β* estimates (figure 4) and correlations between *β* and *π* (see electronic supplementary material, figures S5 and S6). To determine whether these results can be explained by limited data we investigated the practical identifiability of model equation (2.1) for *Netherlands 2016* and *Uganda 2012* data.

#### 3.1.3. Practical identifiability of the within-host model

We investigated whether model equation (2.1) is practically identifiable (see Material and methods) given the two datasets considered—temporal infectious virus titres from birds (wild-caught house sparrows) infected with USUV strains *Netherlands 2016* and *Uganda 2012*. We found that parameters *β* and *π* are weakly practically identifiable for *Netherlands 2016* (table 2A), and not practically identifiable for *Uganda 2012* (table 2B). Parameters *k* and *c* are strongly practically identifiable for both strains (table 2), and *δ* is strongly practically identifiable for *Netherlands 2016* (table 2A), but weakly practically identifiable for *Uganda 2012* (table 2B).

**Table 2.** *Monte Carlo simulation* results for virtual datasets generated at each discrete experimental data point in [11] for (A) *Netherlands 2016* and (B) *Uganda 2012*. AREs give the average relative estimation error for each parameter at noise level *σ,* and *η* determines whether each parameter is strongly (0 < *η* ≤ 1), weakly (1 < *η* ≤ 10) or non-identifiable (*η* > 10).

	*β*	*k*	*δ*	*π*	*c*		*β*	*k*	*δ*	*π*	*c*
parameter	$\frac{\mathrm{ml}}{\mathrm{PFU}\times d}$	$\frac{1}{d}$	$\frac{1}{d}$	$\frac{\mathrm{PFU}}{\mathrm{cell}\times d}$	$\frac{1}{d}$	parameter	$\frac{\mathrm{ml}}{\mathrm{PFU}\times d}$	$\frac{1}{d}$	$\frac{1}{d}$	$\frac{\mathrm{PFU}}{\mathrm{cell}\times d}$	$\frac{1}{d}$
σ=1%						σ=1%					
ARE	1.1	0.4	0.2	1.0	0	ARE	727.7	0.1	4.7	19.0	0.9
*η*	1.1	0.4	0.2	1	0	*η*	727.7	0.1	4.7	19	0.9
σ=5%						σ=5%					
ARE	6.0	3.7	2.9	5.1	0.2	ARE	4027.3	0.7	21.3	86.3	6.1
*η*	1.2	0.74	0.58	1.02	0.04	*η*	805.5	0.14	4.26	17.26	1.22
σ=10%						σ=10%					
ARE	12.6	8.1	7.3	11.6	0.6	ARE	5931.9	1.9	25.6	113.5	9.3
*η*	1.26	0.81	0.73	1.16	0.06	*η*	593.2	0.19	2.56	11.35	0.93
σ=20%						σ=20%					
ARE	24.6	15.2	16.0	27.2	2.3	ARE	5697.9	3.58	24.1	64.4	11.7
*η*	1.23	0.76	0.8	1.36	0.12	*η*	284.9	0.18	1.21	3.22	0.59
(A)						(B)					

Interestingly, parameters *β* and *π* are not identifiable for *Uganda 2012* despite the fact that they are both structurally identifiable (under known initial conditions). We hypothesized that the non-identifiability is due to limited number of birds that have detectable infectious viral titre data [33] (with a single subject having more than two data points above limit of detection). To investigate the dependence of practical identifiability on the number of data points, we repeat the MCS for frequent sample data. We create a synthetic dataset where we assume that two data points were collected each day (every 12 hours, for a total of 14 data points) and compute the new ARE values for these sets. Practical identifiability of *β* and *π* has improved significantly, with the average relative error ARE decreasing for both parameters (table 3) and the within-host model equation (2.1) becoming strongly practically identifiable for both *Netherlands 2016* and *Uganda 2012* (table 3A,B). Note, that this is the minimum number of extra measurements that guarantees practical identifiability of model equation (2.1).

**Table 3.** *Monte Carlo simulation* results for synthetically generated high-frequency datasets containing 14 data points (collected every 12 hours over the first 7 days p.i.) for (A) *Netherlands 2016* and (B) *Uganda 2012*. AREs give the average relative estimation error for each parameter at noise level *σ*, and *η* determines whether each parameter is strongly (0 < *η* ≤ 1), weakly (1 < *η* ≤ 10), or non-identifiable (*η* > 10).

	*β*	*k*	*δ*	*π*	*c*		*β*	*k*	*δ*	*π*	*c*
parameter	$\frac{\mathrm{ml}}{\mathrm{PFU}\times d}$	$\frac{1}{d}$	$\frac{1}{d}$	$\frac{\mathrm{PFU}}{\mathrm{cell}\times d}$	$\frac{1}{d}$	parameter	$\frac{\mathrm{ml}}{\mathrm{PFU}\times d}$	$\frac{1}{d}$	$\frac{1}{d}$	$\frac{\mathrm{PFU}}{\mathrm{cell}\times d}$	$\frac{1}{d}$
σ=1%						σ=1%					
ARE	0.1	0	0	0.1	0	ARE	1.1	0.1	1	0.7	0.3
*η*	0.1	—	—	0.1	—	*η*	1.1	0.1	1	0.7	0.3
σ=5%						σ=5%					
ARE	0.8	0.3	0.3	0.6	0	ARE	5.4	0.4	4.8	2.8	1.7
*η*	0.16	0.06	0.06	0.12	—	*η*	1.08	0.08	0.96	0.56	0.34
σ=10%						σ=10%					
ARE	1.7	0.6	0.6	1.4	0.1	ARE	9.5	0.9	8.1	4.6	2.6
*η*	0.17	0.06	0.06	0.14	0.01	*η*	0.95	0.09	0.81	0.46	0.26
σ=20%						σ=20%					
ARE	3.9	1.6	1.5	3.5	0.3	ARE	17.1	1.8	13.4	6.5	3.4
*η*	0.2	0.08	0.08	0.18	0.15	*η*	0.86	0.09	0.67	0.33	0.17
(A)						(B)					

### 3.2. Bird-to-mosquito transmission scale

Transmission of USUV from an infected bird to a mosquito is dependent on both the infectiousness of the host over age of infection and the probability of contact between a host and a vector. To best describe these interactions we developed three models of probability of host-to-vector transmission equations (2.3)–(2.5) and fitted them to experimental data (see Material and methods).

#### 3.2.1. Dynamics of the bird-to-vector probability of infection functions

**Figure 5.**
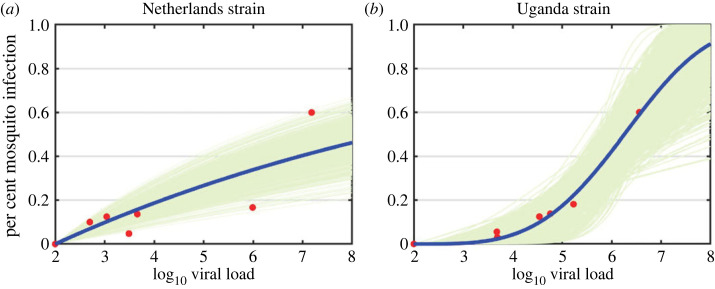
(*a*) Probability of infection function *b*_1_(*V*(*τ*)) found by fitting function equation (2.3) (blue lines) to *Netherlands 2016* per cent mosquito infection data (red dots, measured on $\log_{10}$ scale). (*b*) Probability of infection function *b*_2_(*V*(*τ*)) found by fitting function equation (2.4) (blue lines) to *Uganda 2012* per cent mosquito infection data (red dots, measured on $\log_{10}$ scale). Parameters are given in table 4. Green lines show 1000 fitted trajectories to synthetic data with 46% and 34% relative error.

Based on AIC values, we found that the linear probability of infection function *b*_1_(*V*(*τ*)) best fits the per cent mosquito infection data for *Netherlands 2016* and the power-law probability of infection function *b*_2_(*V*(*τ*)) best fits the per cent mosquito infection data for *Uganda 2012* (figure 5, table 4, electronic supplementary material, figure S7 and table S4). For the resulting *b*_1_(*V*(*τ*)) function for the *Netherlands 2016* strain and *b*_2_(*V*(*τ*)) function for the *Uganda 2012* strain, we computed the relative error, *η*_*i*_, between the predicted probability of infection functions $b_{i}(\log_{10}V)$ and the per cent infected mosquitoes data *b*^*d*^

$$\eta_{i}=\frac{\|b_{i}(\log_{10}(V))-b^{d}{\|}_{2}}{\|b_{i}(\log_{10}(V)){\|}_{2}}\times 100\%,$$

for *i* = {1, 2, 3}. We found that the relative error is $\eta_{1}=46\%$ for $b_{1}(\log_{10}V)$ and *Netherlands 2016* and $\eta_{2}=34\%$ for $b_{2}(\log_{10}V)$ and *Uganda 2012*.

**Table 4.** Parameter estimates for the selected probability of infection functions *b*_1_(*V*(*τ*)) and *b*_2_(*V*(*τ*)) given by fitting equations (2.3) and (2.4) to *Netherlands 2016* and *Uganda 2012* per cent mosquito infection data, respectively. Parameter *a* is measured in $\mathrm{ml}/\log_{10}$ PFU for both *b*_1_(*V*(*τ*)) and *b*_2_(*V*(*τ*)). Parameter *h* is unitless. $\log_{10}\;(\mathrm{LOD})=2$.

	*a*	*h*
*Netherlands 2016*		
$b_{1}=1-\exp(-a(\log_{10}(V)-\log_{10}(\mathrm{LOD})))$	0.10	—
*Uganda 2012*		
$b_{2}=1-\exp(-a{\left(\log_{10}(V)-\log_{10}(\mathrm{LOD})\right)}^{h})$	0.0034	3.67

We next performed parametric bootstrapping [34–36], where we generated 1000 synthetic datasets with the same structure (number of points and identical sampling time) as the original dataset and *η*_*i*_ standard deviation from it. We fitted equations (2.3) and (2.4) to the synthetic data, and the resulting curves (green graphs in figure 5) show an error region for *b*_1_(*V*(*τ*)) and *b*_2_(*V*(*τ*)), respectively.

#### 3.2.2. Practical identifiability of probability of infection functions

To address whether the probability of infection function *b*_1_(*V*(*τ*)) for *Netherlands 2016* and *b*_2_(*V*(*τ*)) for *Uganda 2012* are practically identifiable, we applied the MCS algorithm (see Material and methods) to parameters of models equations (2.3) and (2.4) for virtual datasets obtained by adding noise levels σ={1%,5%,20%} to the seven $\left(\log_{10}v,b^{d}\right)$ data values for the two viral strains [11]. We found that for function *b*_1_(*V*(*τ*)) and *Netherlands 2016* data, parameter *a* is weakly practically identifiable (table 5A). For function *b*_2_(*V*(*τ*)) and *Uganda 2012* data, *a* is unidentifiable and *h* is weakly identifiable (table 5B). As with the within-host model, we expect to improve ARE values by increasing data frequency. Indeed, increasing the number of $\left(\log_{10}v,b^{d}\right)$ data points from 7 to 60 results in strong practical identifiability of *a* for function *b*_1_(*V*(*τ*)) and *Netherlands 2016* data (table 6A). Moreover, it results in weak practical identifiability of *a* and strong practical identifiability of *h* for model *b*_2_(*V*(*τ*)) for *Uganda 2012* data (table 6B).

**Table 5.** *Monte Carlo simulation* results for the best selected probability of infections functions: (A) *b*_1_(*V*(*τ*)) given by equation (2.3) for *Netherlands 2016* per cent mosquito infection data; and (B) *b*_2_(*V*(*τ*)) given by equation (2.4) for *Uganda 2012* per cent mosquito infection data. Parameter *a* is measured in ml/$\log_{10}$ PFU for equations (2.3) and (2.4). Parameter *h* is unitless. AREs give the average relative estimation error for each parameter at noise level *σ*, and *η* determines whether each parameter is strongly (0 < *η* ≤ 1), weakly (1 < *η* ≤ 10) or non-identifiable (*η* > 10).

parameter	*a*	parameter	*a*	*h*
σ=1%		σ=1%		
ARE	1.7	ARE	12.3	2.4
*η*	1.7	*η*	12.3	2.4
σ=5%		σ=5%		
ARE	8.3	ARE	64.3	11.7
*η*	1.66	*η*	12.86	2.34
σ=20%		σ=20%		
ARE	34.3	ARE	607.8	56.4
*η*	1.71	*η*	30.39	2.82
(A)		(B)

**Table 6.** *Monte Carlo simulation* results for data sampled at high-frequency. (A) AREs for parameter *a* obtained from fitting function *b*_1_(*V*(*τ*)) given by equation (2.3) to *Netherlands 2016* per cent mosquito infection data when 60 data points are considered; (B) AREs for parameters *a* and *h* obtained from fitting function *b*_2_(*V*(*τ*)) given by equation (2.4) to *Uganda 2012* per cent mosquito infection data when 60 data points are considered. Parameter *a* is measured in $\mathrm{ml}/\log_{10}$ PFU for equations (2.3) and (2.4). Parameter *h* is unitless. AREs give the average relative estimation error for each parameter at noise level *σ*, and *η* determines whether each parameter is strongly (0 < *η* ≤ 1), weakly (1 < *η* ≤ 10) or non-identifiable (*η* > 10).

parameter	*a*	parameter	*a*	*h*
σ=1%		σ=1%		
ARE	0.5	ARE	3.8	0.7
*η*	0.5	*η*	3.8	0.7
σ=5%		σ=5%		
ARE	2.3	ARE	18.8	3.4
*η*	0.46	*η*	3.76	0.68
σ=20%		σ=20%		
ARE	9.1	ARE	78.4	14
*η*	0.46	*η*	3.92	0.7
(A)		(B)		

### 3.3. Between-host scale

Using an age-structured modelling approach which couples within-host and between-host models, one of our goals was to determine how individual bird USUV infections, combined with bird-to-mosquito probability of infection, influence predictions of USUV incidence in bird and mosquito populations.

#### 3.3.1. Dynamics of the between-host model

**Figure 6.**
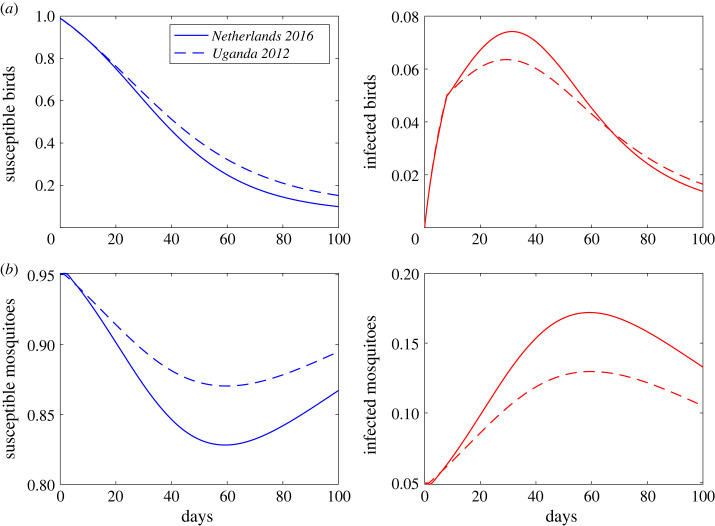
USUV incidence in (*a*) bird populations and (*b*) mosquito populations as given by equation (2.10) and best parameter estimates from fitting *b*_1_(*V*(*τ*)) given by equation (2.3) to *T*_*n*_ and *T*_*u*_ data for *Netherlands 2016* (solid lines) and from fitting *b*_2_(*V*(*τ*)) given by equation (2.4) to *T*_*n*_ and *T*_*u*_ data for *Uganda 2012* data (dashed lines).

We computed the USUV incidence over time in the bird and mosquito populations using a vector-borne SIR model for birds and SI model for mosquitoes (equation (2.10); see Material and methods). To determine differences in disease incidence between the two viral strains, we used transmission functions *β*_*v*_(*τ*) defined by probability of infection functions that best matched the data of each strain, i.e. *b*_1_(*V*(*τ*)) given by equation (2.3) for *Netherlands 2016*, and *b*_2_(*V*(*τ*)) given by equation (2.4) for *Uganda 2012*. We observed similar timing in peak USUV incidence in bird populations, occurring on day 32 for *Netherlands 2016* and on day 30 for *Uganda 2012* (see figure 6*a*, solid versus dashed red lines). Peak USUV incidences in mosquito populations occur 26 and 30 days later than peak incidence in bird populations, for *Netherlands 2016* and *Uganda 2012* strains, respectively (figure 6*b*). *Netherlands 2016* infects more mosquitoes than *Uganda 2012*, 17% compared with 13% (see figure 6*b*, solid versus dashed red lines). Both strains, however, infect similar per cent of birds, 7.5% for *Netherlands 2016* and 6% for *Uganda 2012* (see figure 6*a*, solid versus dashed red lines).

#### 3.3.2. Optimal experimental design

The USUV incidence model equation (2.10) assumed that the probability of bird-to-mosquito infection functions have fundamentally different characteristics for the two virus strains considered. More specifically, it assumed that the per cent mosquito infection increases linearly with the $\log_{10}$ infectious viral load for *Netherlands 2016*, and super-linearly with the $\log_{10}$ infectious viral load (power coefficient *h* = 3.67) for *Uganda 2012*. Since we do not expect that the shape of the bird-to-mosquito infection functions vary by strain, we investigated how the strain-specific disease incidence predictions change when the other two functions (that did not result in a best fit) were considered: *b*_2_(*V*(*τ*)), *b*_3_(*V*(*τ*)) for *Netherlands 2016* and *b*_1_(*V*(*τ*)), *b*_3_(*V*(*τ*)) for *Uganda 2012*. We obtained large variability between predicted USUV incidence in both bird and mosquito populations for both strains (see electronic supplementary material, figure S8). We investigate whether an improved initial empirical dataset will create more robust parameter values in the probability of infection functions, and potentially unify results at the between-host scale. For example, it would be useful to know whether there is a viral load threshold where all mosquitoes get infected, or (alternatively) there is a viral load threshold where mosquito infection levels off. We performed two *in silico* optimal experimental designs: hypothesis 1 assumed that the viral load where all mosquitoes get infected is known, and hypothesis 2 assumed that per cent mosquito infection levels off at 60% (see electronic supplementary material). We fitted the probability of infection functions equations (2.3)–(2.5) to these datasets (see electronic supplementary material, figures S9 and S10) and found that the power-law function *b*_2_(*V*(*τ*)) becomes the best fit (lowest AIC) for both USUV strains in both scenarios (see electronic supplementary material, tables S7 and S8). This suggests that optimal experimental design together with frequent data measurements is needed to improve predictions.

## 4. Discussion

Understanding the epidemiology of emerging pathogens, such as USUV, requires systems investigation at each scale involved in the host–virus transmission cycle, from individual infection in birds, to bird-to-vector transmission, to USUV incidence in bird and vector populations and eventually to spillover probability in humans [37–39]. For new pathogens, field data is sparse, and predictions are based on laboratory-type inoculation and transmission experiments [9,11] combined with dynamical mathematical modelling [9].

In this study, we developed an age-structured within-between-host modelling approach for investigating the differences in the dynamics of two USUV strains, *Netherlands 2016* of European descent and *Uganda 2012* of African descent, in bird and mosquito populations. Our study investigated the USUV dynamics at three scales: the within-host scale, where the dynamics of individual bird infection were considered; bird-to-mosquito transmission scale, where the per cent of mosquito infections based on virus level was considered; and between-host scale, where a vector-borne age-structured epidemiological model of bird and mosquito interactions was considered. The models developed for the first two scales were validated against laboratory-produced infectious viral titre data from individual bird inoculation studies [11] and against per cent mosquito infection data [11]. The last scale had no empirical data for validation. Instead, we used results from the first two scales to make predictions for the dynamics of USUV incidence in bird and mosquito populations.

To determine differences in USUV strain dynamics in individual bird infections (within-host scale), we validated a target cell limited within-host model with eclipse phase equation (2.1) against longitudinal infectious USUV data in wild-caught house sparrows inoculated with either *Netherlands 2016* or *Uganda 2012* [11]. Before performing data fitting, we conducted structural identifiability analyses which showed that, if the number of target cells is not known, it is not possible to estimate the rate at which infected cells produce new viral particles each day. When, however, the initial target cell concentration and initial viral load are known, all within-host model parameters can be uniquely determined from unlimited, noise-free viral load observations [24,26,33]. Consequently, we estimated the model parameters using a nonlinear mixed effects approach, and observed heterogeneity in outcomes at both within- and between-strain levels (figure 3). Moreover, we observed correlations between infection rate *β* and viral shedding rate *π*, especially for *Uganda 2012*, for which data were sparse and the number of subjects was low (figure 4, electronic supplementary material, figures S5 and S6). These correlations occurred despite our findings that all parameters are globally structurally identifiable (under unlimited data and known initial conditions). We attributed these results to lack of practical identifiability, given the limited experimental data available. Using practical identifiability analyses, we determined that a minimum of 14 infectious virus titre measurements collected every 12 hours (double the initial amount) are needed to achieve practical identifiability of all within-host model parameters (table 3). This experimental design can be achieved by either sampling each bird twice a day or by creating two cohorts with daily morning or daily evening samples and then fitting models to the entire population data. We anticipate that this key result will serve as a guideline for the experimental designing of other within-host inoculation studies.

To determine differences in bird-to-mosquito USUV transmission for the two viral strains, we developed three probability of infection functions that establish a relationship between the infectious virus titre inside a bird and the per cent of mosquito infection upon contact: the linear model *b*_1_(*V*(*τ*)) given by equation (2.3), the power-law model *b*_2_(*V*(*τ*)) given by equation (2.4) and the density-dependent model *b*_3_(*V*(*τ*)) given by equation (2.5) [19]. Data fitting determined that the linear model *b*_1_(*V*(*τ*)) best fits the *Netherlands 2016* data and the power-law model *b*_2_(*V*(*τ*)) best fits the *Uganda 2012* data (table 4 and electronic supplementary material, table S4). This means that while the per cent mosquito infection grows linearly with the virus load within an infected bird (on $\log_{10}$ scale) for *Netherlands 2016*, it grows super-linearly (power coefficient *h* = 3.67) with the virus load within an infected bird (on $\log_{10}$ scale) for *Uganda 2012*. Biologically, this suggests that the per cent mosquito infections is proportional to the viral load inside the bird they feed on for *Netherlands 2016* infections. By contrast, the per cent of mosquito infections is low when the viral load inside the bird they feed on is low and grows exponentially when the viral load inside the bird they feed on is high for *Uganda 2012* infections. As with the within-host scale, we investigated the practical identifiability of the selected probability of infection functions and found that, for both viral strains, none of the parameters are strongly identifiable (table 5). This outcome is not surprising considering that only seven birds were used in this experiment and, consequently, only seven data points were used for data fitting. We investigated whether practical identifiability improves under abundant data by creating *in silico* experiments with increased number of measurements and fitting the probability of infection functions to them. We found that, unlike the within-host model, improving parameter identifiability under addition of data is not straightforward. Even an increase of *in silico* measurements to 60 (from the original seven), did not result in strong practical identifiability for the power-law model and *Uganda 2012* data (table 6).

The ultimate goal of our study was to use the outcomes of the within-host scale and bird-to-mosquito transmission scale (obtained from validating them against data at these biological scales) to make predictions at the between-host scale (for which we have no *a priori* data) when *Netherlands 2016* or *Uganda 2012* strains are predominant in the wild. We developed a vector-borne age-structured within-between-host model (equation (2.10)) which assumed that the mosquito infectivity rate is dependent on the probability of vector infection given by function *b*_1_(*V*(*τ*)) (equation (2.3)) for *Netherlands 2016* and function *b*_2_(*V*(*τ*)) (equation (2.4)) for *Uganda 2012*. We compared the USUV incidence predictions for these selected (lowest AIC) probability of infection functions and found similar peak disease incidence in birds regardless of the infectious strain and 4% higher peak disease incidence in mosquitoes when *Netherlands 2016* (rather than *Uganda 2012*) was the infectious strain considered.

The transmission scale results predicted that the probability of bird-to-mosquito infection functions have fundamentally different characteristics for the two virus strains, with per cent mosquito infection growing linearly with the $\log_{10}$ infectious viral load for *Netherlands 2016*, and super-linearly with the $\log_{10}$ infectious viral load (power coefficient *h* = 3.67) for *Uganda 2012*. Since we did not expect that the shape of the bird-to-mosquito infection function would vary by strain, we investigated whether knowledge of the long-term per cent mosquito infection can alter these results. Hence, we considered two *in silico* experimental design studies: one in which we assume known the viral load threshold needed for the entire mosquito population to get infected (hypothesis 1 in the electronic supplementary material) and one in which we assume that the per cent mosquito infection levels off at 60% (hypothesis 2 in the electronic supplementary material). Fitting to both these *in silico* datasets held the power-law transmission function as the best fit for both *in silico* viral strains (electronic supplementary material, tables S7 and S8). In future experimental studies, we aim to determine the relationship between per cent mosquito infection and viral load by designing artificial bloodmeal experiments, where mosquitoes are exposed to cotton balls containing blood of increased concentrations of *Netherlands 2016* and *Uganda 2012* virus, respectively. We have previously conducted such experiments (for a fixed inoculum) when establishing that *C. quinquefasciatus* mosquitoes are susceptible to USUV [11].

Our study has several limitations. First, the results of the within-host scale predict heterogeneity in virus dynamics between cohorts and within birds of a cohort (including large variability in predicted *R*_0_ estimates among the birds infected with the *Uganda 2012* strain). As explained above, some of this is due to uncertainty in model fitting, which can be improved under optimal experimental design where more data measurements (twice a day) and more birds are included. Another layer of uncertainty comes from the fact that the birds were inoculated, rather than infected by mosquitoes. To compensate for that, we considered a lower initial viral load (than the maximum inoculum) when determining parameter estimates in the within-host model. An even lower viral load may initiate the infection (in the wild), which may result in shorter incubation rates, and/or delayed peak viremia. Inoculation studies with varied inoculum dose would be needed to determine the role the inoculum plays in virus dynamics and disease outcomes.

Second, we assumed that all exposed mosquitoes get infected in the presence of high viral titres inside the host they feed on. That may not be realistic, and, adjustments to the probability of infection functions may be needed to account for a lower maximal probability of infection. Moreover, in the age-structured epidemiological model we assumed that an infected mosquito immediately finds an array of hosts to infect. While that may be correct in the laboratory setting, it is not the case in the wild. Third, we assumed fixed mosquito-to-bird contact rates and constant mosquito-to-bird transmission probabilities (regardless of viral dynamics inside an infected mosquito). Relaxing these assumptions may change the current results. Fourth, the use of a limited number of birds and the use of only two bird species (wild-caught house sparrows and juvenile chickens) is another limitation that may prevent us from generalizing the results to other bird populations. Last, we assumed that all parameters are lognormally distributed during data fitting and all noise levels are normally distributed when conducting practical identifiability analyses. Previous studies have shown that the degree of uncertainty in parameter estimates vary for the different noise distributions and estimation methods [40], so our results are limited to our assumptions of distributions.

In conclusion, we investigated the dynamics of two strains of USUV at the within-host scale, bird-to-vector transmission scale and vector-borne epidemiological scale. We used individual within-host infectious virus data and per cent mosquito infection data to predict USUV incidence in bird and mosquito populations. We addressed the role of model structure, data uncertainty and optimal experimental design on model predictions. We found that uncertainty in predictions at one scale may change predicted results at another scale. Optimal experimental design was proposed, suggesting that increased data frequency improves predictions.

## Data Availability

Data and relevant code for this research work are stored in GitHub: www.github.com/NecibeTuncer/Usutu and have been archived within the Zenodo repository: https://zenodo.org/records/10493294 [41]. Supplementary material is available online [42].
